# JAM-A: Adhesion Receptor and Signaling Regulator in Atherosclerosis

**DOI:** 10.1007/s11883-025-01322-x

**Published:** 2025-07-29

**Authors:** Mariel F. Schwietzer, Klaus Ebnet

**Affiliations:** 1 Molecular Cardiology, Cardiology I, Medical Clinic C, University Hospital Münster, 48419 Münster, Germany; 2 Institute-associated Research Group “Cell adhesion and cell polarity”, Institute of Medical Biochemistry, ZMBE, University of Münster, 48419 Münster, Germany

**Keywords:** Atherosclerosis, Coronary artery disease, JAM-A, Leukocyte-endothelial cell interaction, Platelet aggregation, Thrombosis

## Abstract

**Purpose of Review:**

Cell-cell adhesion between leukocytes, platelets and endothelial cells plays a critical role in vascular inflammation and thrombus formation. This review aims at providing a comprehensive picture of the contribution of the immunoglobulin superfamily (IgSF) cell adhesion receptor Junctional Adhesion Molecule-A (JAM-A) to the process of atherosclerosis.

**Recent Findings:**

Proinflammatory and proatherogenic stimulation of endothelial cells results in redistribution of JAM-A from cell-cell junctions to the apical surface to promote monocyte adhesion and transmigration. Agonist-stimulation of platelets results in elevated surface levels of JAM-A concomitant with enhanced release of soluble JAM-A (sJAM-A). sJAM-A promotes platelet aggregation, thrombus formation, and platelet-monocyte aggregate formation. Elevated levels of sJAM-A correlate with recurrent myocardial infarction.

**Summary:**

JAM-A is expressed by several cell types implicated in atherogenesis, notably endothelial cells, platelets, and leukocytes. Proinflammatory and proatherogenic stimuli induce a redistribution of JAM-A within endothelial cells. Stimulated platelets release sJAM-A into the circulation. This review illustrates the role of JAM-A in atherogenesis and elaborates the underlying mechanisms.

## Introduction

Atherosclerosis is a chronic inflammatory disease of the arterial wall [1, 2]. It is initiated by structural damage of the endothelial layer and the retention of lipoproteins at the subendothelial space. Lipid deposition triggers the accumulation of monocyte-derived macrophages in the intima, thereby inducing a low-grade inflammatory response. The resulting activation of endothelial cells (EC) initiates the accumulation of additional leukocytes as well as the migration and proliferation of vascular smooth muscle cells (SMC), which eventually results in the formation of an atherosclerotic plaque. Plaques are usually covered by a thick fibrous cap which shields the prothrombotic components in the plaque from the cellular and soluble blood components (stable plaque). However, the fibrous cap may erode over time resulting in a thin cap that is susceptible to rupture (vulnerable plaque). A small gap in the fibrous cap (plaque rupture) can expose the highly thrombogenic core to the blood which leads to blood coagulation and thrombus formation and eventually vessel occlusion [3].

The recruitment of leukocytes, platelets and SMCs to the atherosclerotic plaque is regulated by a plethora of cell adhesion receptors [4]. For example, leukocytes use selectins, selectin ligands, and integrins to interact with endothelial-expressed selectin ligands, selectins, and members of the IgSF, respectively, to bind to the apical membrane of EC as prerequisite of their extravasation into the subendothelial space [4]. Similarly, platelets express members of all three families of adhesion molecules, i.e. selectins, integrins and IgSF members, to interact with each other, with extracellular matrix (ECM) proteins and fibrinogen, and with leukocytes and EC [5]. Finally, activated EC upregulate and partially relocalize various adhesion molecules to make them available for adhesion and transendothelial migration of leukocytes [6, 7].

JAM-A is a type I transmembrane protein with two extracellular Ig-like domains [8]. It is the founding member of the junctional adhesion molecule (JAM) family which consists of JAM-A, JAM-B and JAM-C [9]. It has originally been identified as a surface receptor on platelets [10] but is expressed by many cell types including various leukocyte subsets, EC, and vascular SMCs [11–13]. Its extracellular domain mediates a trans-homophilic interaction, a mechanism that regulates its enrichment at sites of cell-cell contacts. Through heterophilic interaction in trans with the leukocyte integrin αLβ2 JAM-A supports interactions of JAM-A-expressing cells with leukocytes. Through heterophilic interactions in cis with integrins expressed on EC and tumour cells, i.e. αVβ3 and αVβ5 integrins, and with integrins expressed on platelets, i.e. αIIbβ3 integrin, it forms signaling complexes which regulate integrin-mediated processes [9, 14].

As opposed to many other cell adhesion receptors with two Ig-like domains, the trans-homophilic activity of JAM-A is not strong enough to mediate cell aggregation when expressed in a heterologous cell system. Homophilic JAM-A interactions therefore primarily serve to recruit cytoplasmic adapters and signaling molecules to sites of cell-cell contacts rather than to physically strengthen cell-cell adhesion. In fact, JAM-A has been described to regulate the activity of a number of signaling molecules and pathways including Rho and Ras family small GTPases [14–18], PI(3)K – Akt signaling [16, 19], the Hippo pathway [20], the Erk1/2 MAPK signaling pathway [14, 21] and the activity of Src kinase [14, 22].

### A role for JAM-A in Atherosclerosis

As outlined above, JAM-A is expressed by basically all cell types involved in atherosclerotic plaque formation, i.e. EC, SMC, monocytes/macrophages and platelets. Initial evidence for a role of JAM-A in atherogenesis was provided by studies which demonstrated that JAM-A is upregulated in early atherosclerotic endothelium of carotid arteries of apolipoprotein E (apoE)-deficient mice, and which showed that soluble JAM-A (sJAM-A) inhibited the binding of monocytes and memory T cells to the atherosclerotic endothelium [23, 24]. Complementary findings revealed that genetic deletion of JAM-A in atherosclerosis-prone apoE^−/−^ mice reduced neointimal hyperplasia, decreased neointimal macrophage content, and impaired the adhesion of monocytes to ex vivo perfused carotid arteries [25]. A clinical study demonstrated a positive correlation of sJAM-A with the severity of coronary artery disease in humans [26]. Together, these observations strongly suggested a role of JAM-A in atherogenesis, a concept that has been reinforced by subsequent studies. For example, a peptide designed to block trans-homophilic JAM-A interactions reduced the number of aortic lesions in apoE^−/−^ mice and the number of macrophages and SMC present within the atherosclerotic plaques [27, 28]. In addition, intravenous administration of exosomes containing microRNA-145 (miR-145), which has previously been shown to target human JAM-A [29], reduces plaque formation in apoE^−/−^ mice [30]. Another miRNA that targets JAM-A and that reduces monocyte adhesion to stimulated EC, i.e. the plant-derived miR-156a, is abundantly present in normal human serum but significantly reduced in serum of CVD patients [31]. These observations are consistent with the notion that high levels of JAM-A promote atherosclerosis. However, they cannot provide information on the mechanism by which JAM-A promotes atherosclerosis. This is partly owing to the fact that JAM-A is expressed in virtually all cell types involved in atherosclerosis. It is therefore important to delineate the cell type-specific functions of JAM-A in the context of atherogenesis.

### JAM-A in Endothelial Cells

In multicellular tissues such as epithelia and endothelia JAM-A is localized at cell-cell junctions [11]. Stimulation of EC with a cocktail of the proinflammatory cytokines TNF-α and IFN-γ, or with the chemokine ligand 2 (CCL2) triggers a redistribution of JAM-A from intercellular junctions to the apical membrane domain, which correlates with increased JAM-A-mediated adhesion of various leukocyte subsets [32–34]. Importantly, JAM-A redistributes from intercellular junctions in response to altered flow conditions [35], which is present at sites of vessel bifurcations where atherosclerotic plaque formation prevails [3]. As pointed out above, JAM-A interacts with the leukocyte-specific αLβ2 integrin which is expressed by many leukocyte subsets including monocytes and macrophages [33]. The JAM-A – αLβ2 interaction is physically stronger than the homophilic JAM-A – JAM-A interaction [36] suggesting that JAM-A can directly contribute to leukocyte recruitment to sites of inflammation. This function of JAM-A is most likely important at the level of leukocyte adhesion but also at the level of leukocyte transendothelial migration, as both processes can be blocked by antibodies against JAM-A [33]. Endothelial JAM-A also mediates adhesion of platelets to activated EC [37] suggesting a potential contribution to thrombus formation.

There is strong evidence that endothelial JAM-A contributes to atherosclerosis by promoting leukocyte recruitment. Immunofluorescence analyses and molecular ultrasound imaging of JAM-A indicated that JAM-A is upregulated in regions of altered vascular blood flow and redistributes from interendothelial junctions to the apical surface of EC [13, 24, 38]. Similar to the pro-inflammatory cytokines TNF-α and IFN-γ, pro-atherogenic oxidized low density lipoprotein (oxLDL) causes a redistribution of JAM-A from junctions to the apical surface, which is associated with increased monocyte transendothelial migration [39]. Similarly, apolipoprotein C3 (APOC3), a protein that is found in many lipoprotein particles and is associated with cardiovascular disease (CVD) [40] enhances JAM-A expression in EC and increases monocyte adhesion to EC [41]. Thus, conditions known to promote atherosclerosis upregulate JAM-A expression, induce its relocalization to the apical membrane, and promote monocyte adhesion. Importantly, JAM-A expression is significantly enriched in vulnerable compared to stable atherosclerotic plaques [13], suggesting that JAM-A could serve as a prognostic marker for identifying high-risk atherosclerotic lesions. Mice with a 50% reduction of JAM-A expression specifically in EC have less atherosclerotic lesions and reduced necrotic core size [35], a hallmark of advanced lesions. These mice also had a lower number of monocytes in the subendothelial area, probably due to impaired transmigration activity caused by the JAM-A-depleted endothelium in situ [35]. These findings clearly indicate a pro-atherogenic role of EC-expressed JAM-A, which is most likely based on its ability to promote monocyte recruitment to the atherosclerotic lesions through its interaction with αLβ2 integrin and perhaps JAM-A on monocytes.

### JAM-A in Platelet Function

JAM-A has been identified as a platelet surface molecule recognized by an antibody which stimulates platelet aggregation and fibrinogen binding, as well as protein kinase C (PKC) membrane translocation and activation [10, 42, 43]. These early observations implicated a signaling role of JAM-A in platelets which regulates a critical aspect of platelet physiology and function, i.e. platelet aggregation and thrombus formation. More recent findings have revealed a inhibitory role of JAM-A in platelet functions. In constitutive JAM-A knockout mice, platelets spread and aggregate faster, and a number of platelet-mediated physiological processes including bleeding time, thrombus formation, vessel occlusion, and clot retraction are enhanced or accelerated [44]. The underling mechanism of this inhibitory function of JAM-A is based on its lateral, i.e. cis-association with platelet integrin αIIbβ3 and a direct interaction of its cytoplasmic domain with C-terminal Src kinase (Csk) [22]. This lateral association can bring JAM-A-bound Csk in close spatial proximity with αIIbβ3 integrin-associated c-Src kinase [45], allowing inhibition of c-Src through phosphorylation at its inhibitory tyrosine residue 530 [22]. A loss of JAM-A, thus, releases αIIbβ3 integrin-associated Src from Csk-mediated inhibition and triggers signaling pathways downstream of active Src including pathways which regulate granule secretion, platelet spreading, and platelet aggregation [46, 47].

Studies with platelet-specific conditional JAM-A KO mice support an inhibitory function of JAM-A on platelet activity and a protective role of platelet-expressed JAM-A in atherosclerosis. In the absence of platelet-expressed JAM-A in apoE^−/−^ mice, aortic plaque formation is increased, associated with augmented numbers of infiltrating immune cells including macrophages and T cells as well as SMCs, enhanced platelet coverage on the injured endothelium, and increased adhesion of monocytic cells to platelets [48, 49]. Thus, in contrast to the atherosclerosis-promoting role of EC-expressed JAM-A, platelet-expressed JAM-A has an atherosclerosis-inhibiting role. This function is at least in part based on its lateral association with αIIbβ3 integrin and its cytoplasmic association with Csk, which serves to limit αIIbβ3 integrin-mediated outside-in signaling via inhibition of Src kinase.

However, this represents only one aspect of the multiple roles of JAM-A in platelet physiology and pathophysiology. As pointed out above, the levels of sJAM-A correlate with the severity of coronary artery disease in humans [26]. It has previously been shown that JAM-A is released from EC by A Disintegrin And Metalloprotease (ADAM) 10 and 17, whose activity is enhanced by TNF-α and IFN-γ as well as by platelet activating factor (PAF) [50]. A recent study shows that JAM-A is also released from activated platelets, in part associated with the membrane of microparticles [51]. This study also showed that sJAM-A cooperates with various platelet agonists in platelet activation, aggregation and thrombus formation by interacting with surface-localized JAM-A on platelets. sJAM-A also induces clearance of platelets by monocytes thereby promoting monocyte differentiation into macrophages and foam cells [51]. Of note, high serum levels of sJAM-A are associated with thrombo-ischemic complications in coronary artery disease (CAD) and poorer prognosis for recurrent myocardial infarctions. Importantly, the cooperative activity of sJAM-A with platelet agonists is mediated by a trans-homophilic interaction between sJAM-A and membrane-bound JAM-A on platelets indicating that the homophilic JAM-A interaction transmits co-stimulatory signals to platelet activation. It is likely that sJAM-A released by various cell types including activated platelets, inflammatory EC and leukocytes [50, 52] accumulates in the circulation to levels that override the intrinsic atheroprotective effect of membrane-bound JAM-A.

### JAM-A in Monocytes/Macrophages in Atherosclerosis

JAM-A is expressed by various leukocyte subsets including monocytes and macrophages [53–55]. Studies with JAM-A-deleted polymorphonuclear cells (PMNs) showed that the lack of JAM-A in these cells results in an impaired extravasation to inflammatory sites due to increased adhesion to the endothelium. This has been interpreted as an inability of PMNs to de-adhere from EC [56]. Similar observations have been reported for monocytes. JAM-A-deficient monocytes adhere more strongly to EC and fail to complete transendothelial migration [35]. In atherogenic mice, the absence of JAM-A in monocytes leads to an overall increase in plaque areas despite reduced macrophage contents in atherosclerotic lesions [35]. The increase in plaque areas is most likely due to indirect effects such as platelet interaction with monocytes or altered cytokine release rather than enhanced macrophage infiltration. Interestingly, activated macrophages phagocytose microparticles coated with anti-JAM-A antibodies [13], suggesting that JAM-A-mediated signals enhance macrophage phagocytic activity and promote their differentiation into foam cells.

### JAM-A in Smooth Muscle Cells in Atherosclerosis

As compared to EC and platelets, the contribution of SMC-expressed JAM-A to atherosclerosis has not been studied in great detail yet. However, JAM-A is expressed in cultured SMC and its expression is enhanced by pro-inflammatory cytokines [12, 13]. An accumulation of JAM-A-expressing SMC in the intima of human atherosclerotic arteries has been documented [12]. A JAM-A blocking peptide reduced intima hyperplasia in a mouse model of carotid artery stenosis [57]. Altogether, these observation suggest a contribution of SMC-expressed JAM-A to atherosclerotic plaque formation.

### Mechanistic Insights: JAM-A in Atherosclerosis

As mentioned above, the trans-homophilic activity of JAM-A is rather weak as it does not support cell aggregation or cell adhesion to immobilized recombinant JAM-A-Fc fusion proteins when expressed in a heterologous cell system [58]. It rather serves to enrich JAM-A at sites of cell-cell interactions [59, 60]. The trans-heterophilic interaction of JAM-A with αLβ2 integrin is stronger than the trans-homophilic JAM-A interaction [36]. Therefore, many observations showing a role of JAM-A in inflammation and atherosclerosis cannot be explained by a direct contribution of JAM-A to the physical strength of cell-cell interactions.

The lateral association of JAM-A (cis-interaction) with integrins could be one mechanism by which JAM-A contributes to atherosclerosis. In EC, JAM-A exists in a ternary complex with the tetraspanin (Tspan) family member CD9 and the integrin family member αVβ3 integrin [21, 61]. In this complex, CD9 links serves as a linker between JAM-A and αVβ3 integrin [21]. RGDS peptide, which mimics ligand binding and integrin activation, triggers increased association of αVβ3 integrin with JAM-A [61], suggesting that integrin activation promotes the formation of the ternary complex. Importantly, basic fibroblast growth factor, a pro-angiogenic agonist in EC which activates the Erk1/2 MAPK signaling pathway [62], dissociates the ternary complex, suggesting that JAM-A inhibits bFGF signaling [21, 61]. A similar complex has been identified in platelets, where JAM-A is associated with αIIbβ3 integrin and suppresses αIIbβ3 integrin-mediated outside-insignaling [22, 44]. As described above, Csk recruited by JAM-A limits the activity of Src kinase associated with αIIbβ3 integrin [22], providing a mechanistic example of how JAM-A can modulate integrin-based signaling processes. Of note, similar complexes have been identified in other cells. In tumour cells, JAM-A interacts with αVβ5 integrin via Tspans CD9 and CD81 and limits αVβ5 integrin-mediated signaling and cell motility [14]. In polarized epithelial cells, JAM-A interacts with α3β1 integrin via Tspans CD9 and CD151 and restricts the speed of single cell migration [63]. Taken together, these findings support a general concept that cis-interactions between JAM-A and integrins attenuate integrin activation and integrin-mediated signaling pathways.

How could this concept be transferred to the role of JAM-A in atherogenesis? As outlined above, atherosclerotic diseases are associated with increased levels of sJAM-A. The interaction of sJAM-A with platelet-expressed JAM-A could possibly disrupt the JAM-A – αIIbβ3 complex thereby releasing αIIbβ3 from inhibition by JAM-A [22]. More recently, it was described that JAM-A-mediated interaction of platelets with surface-immobilized JAM-A occurs only in the presence of co-immobilized fibrinogen, a major ligand of αIIbβ3 integrin [64, 65]. It is not clear whether a similar inhibitory cis-interaction of JAM-A with αLβ2 integrin exists in monocytes. However, the original identification of αLβ2 integrin as an interaction partner for JAM-A was based on a yeast two-hybrid screening experiment [33] which opens the possibility of an inhibitory cis-interaction of JAM-A and αLβ2 integrin. Increased levels of sJAM-A under atherosclerotic conditions could release membrane-bound, inhibitory JAM-A from αLβ2 integrin. This could explain the impaired ability of JAM-A-deficient monocytes to de-adhere from EC and, as a consequence, an increase in lesions in atherosclerotic-prone mice due to enhanced recruitment of platelets and possibly their phagocytosis by activated macrophages [39].

## Conclusions

**Fig. 1:**
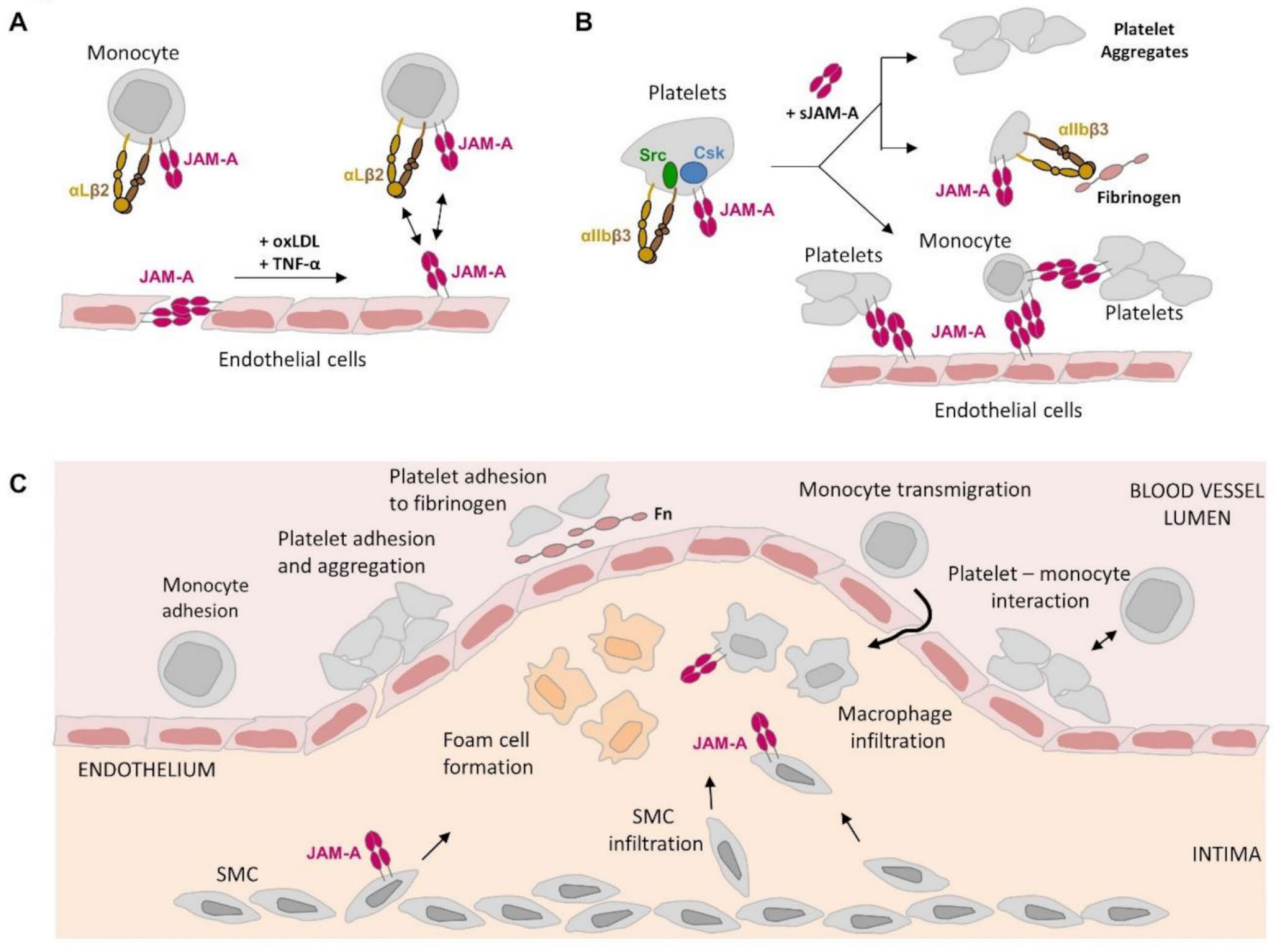
Putative function of JAM-A in different cell types during atherogenesis. (**A**) JAM-A function in endothelial cells. JAM-A is normally localized at interendothelial junctions but is relocalized to the apical membrane domain in response oxLDL and pro-inflammatory cytokines. Apically localized JAM-A interacts with αLβ2 integrin and JAM-A on monocytes. (**B**) JAM-A function in platelets. JAM-A associates with αIIbβ3 integrin in cis and limits the activity of αIIbβ3 integrin-associated Src kinases through Csk. Soluble JAM-A released from platelets and endothelial cells activates platelet functions including aggregation and αIIbβ3 integrin-mediated binding to fibrinogen. Activated platelets also interact with endothelial cells and endothelial cell-bound monocytes. (**C**) JAM-A expression in plaque-associated cells. JAM-A is expressed by macrophages in the atherosclerotic plaque. Since JAM-A stimulates macrophage phagocytic activity it might contribute to the formation of foam cells through JAM-A-triggered phagocytosis of platelets. JAM-A expression by smooth muscle cells contributes to plaque formation by regulating smooth muscle cell migration. Abbreviations: Fn, fibrinogen; oxLDL, oxidized low density lipoprotein; sJAM-A, soluble JAM-A; SMC, smooth muscle cell

Evidence accumulating in recent years strongly supports a role for JAM-A during atherosclerosis. JAM-A is expressed by virtually all cell types involved in atherogenesis including EC, platelets, monocytes/macrophages and SMCs (Fig. 1). Except for platelets, reducing JAM-A expression ameliorates atherogenesis in atherosclerotic-prone mice, which is indicative of a pro-atherogenic function of JAM-A. The atheroprotective role of platelet-expressed JAM-A is most likely offset by the atherogenesis-promoting function of sJAM-A released from EC, platelets and possibly other cells types. Importantly, plasma levels of sJAM-A as well as JAM-A surface levels on platelets are predictive for future outcomes of coronary artery disease [51]. In addition, elevated JAM-A expression in vulnerable versus stable atherosclerotic plaques suggests its potential as a marker of plaque instability—a critical determinant of plaque rupture and major cardiovascular complications such as myocardial infarction and stroke [13]. JAM-A expression levels may thus serve as a prognostic marker for disease development. In the future, it will become an important task to reveal the molecular mechanisms underlying JAM-A’s atherogenic function. It will be of particular interest to understand the crosstalk between JAM-A and integrins in each cell type. Insights into theese molecular mechanisms may facilitate the design of interventions to interfere with JAM-A–mediated extracellular binding or intracellular signaling, thereby offering novel approaches to mitigate its pro-atherogenic role in coronary artery disease.

### Key References

• 13. Zhang YJ, Bai DN, Du JX, Jin L, Ma J, Yang JL, et al. Ultrasound-guided imaging of junctional adhesion molecule-A-targeted microbubbles identifies vulnerable plaque in rabbits. Biomaterials. 2016;94:20–30. 10.1016/j.biomaterials.2016.03.049.

This study provides evidence that JAM-A-expressing cells are preferentially present in vulnerable plaques in a rabbit model of atherosclerosis. The study also shows that microbubbles coated with anti-JAM-A antibodies are phagocytosed by activated macrophages indicating that macrophage-expressed JAM-A triggers phagocytic activity which may contribute to the engulfment of JAM-A-expressing platelets and foam cell formation.

•• 22. Naik MU, Caplan JL, Naik UP. Junctional adhesion molecule-A suppresses platelet integrin alphaIIbbeta3 signaling by recruiting Csk to the integrin-c-Src complex. Blood. 2014;123(9):1393 − 402. 10.1182/blood-2013-04-496232.

This study describes a molecular mechanism through which JAM-A expressed on platelets limits the activity of αIIbβ3 integrin. It shows that JAM-A is associated with αIIbβ3 integrin and that JAM-A-bound Csk limits the activity of αIIbβ3 integrin-bound Src family kinases. The hyperreactivity of platelets observed after depletion of JAM-A can be explained by an uncontrolled αIIbβ3 integrin-mediated outside-in signaling. This study provides important insights into the thrombosis-protective role of JAM-A.

• 26. Cavusoglu E, Kornecki E, Sobocka MB, Babinska A, Ehrlich YH, Chopra V, et al. Association of plasma levels of F11 receptor/junctional adhesion molecule-A (F11R/JAM-A) with human atherosclerosis. Journal of the American College of Cardiology. 2007;50(18):1768-76. 10.1016/j.jacc.2007.05.051.

This study provides strong evidence that the plasma levels of JAM-A correlates with the severity of coronary artery disease in humans.

• 28. Babinska A, Clement CC, Przygodzki T, Talar M, Li Y, Braun M, et al. A peptide antagonist of F11R/JAM-A reduces plaque formation and prolongs survival in an animal model of atherosclerosis. Atherosclerosis. 2019;284:92–101. 10.1016/j.atherosclerosis.2019.02.014.

This study shows that a synthetic peptide that blocks the trans-homophilic interaction of JAM-A limits platelet adhesion to endothelial cells, reduces plaque formation and prolongs survival in atherosclerotic-prone mice. Interfering with JAM-A function may positively influence coronary artery disease in humans.

•• 33. Ostermann G, Weber KS, Zernecke A, Schroder A, Weber C. JAM-1 is a ligand of the beta(2) integrin LFA-1 involved in transendothelial migration of leukocytes. Nat Immunol. 2002;3(2):151-8. 10.1038/ni755.

This study identifies the leukocyte-expressed αLβ2 integrin as a trans-heterophilic interaction partner of JAM-A. The study also provides evidence that this interaction occurs subsequent to the initial transient interactions (rolling) of leukocyte interactions with endothelial cells and and regulates their adhesion and transmigration. The findings also suggest a potential cis-interaction of JAM-A and αLβ2 integrin in leukocytes.

•• 35. Schmitt MM, Megens RT, Zernecke A, Bidzhekov K, van den Akker NM, Rademakers T, et al. Endothelial junctional adhesion molecule-a guides monocytes into flow-dependent predilection sites of atherosclerosis. Circulation. 2014;129(1):66–76. 10.1161/CIRCULATIONAHA.113.004149.

By comparing somatic JAM-A^−/−^ mice, endothelial-specific JAM-A^−/−^ mice and leukocyte-specific JAM-A^−/−^ mice, this study dissects the contributions of endothelial-expressed JAM-A and leukocyte-expressed JAM-A to atherosclerosis. The study also provides evidence that JAM-A-deficient monocytes have a defect in disconnecting from endothelial cells during extravasation.

•• 51. Rath D, Rapp V, Schwartz J, Winter S, Emschermann F, Arnold D, et al. Homophilic Interaction Between Transmembrane-JAM-A and Soluble JAM-A Regulates Thrombo-Inflammation: Implications for Coronary Artery Disease. JACC Basic Transl Sci. 2022;7(5):445 − 61. 10.1016/j.jacbts.2022.03.003.

The study shows that platelets release JAM-A and that soluble JAM-A interacts with membrane-bound JAM-A on platelets. This triggers a signal which cooperates with platelet agonists in the stimulation of signaling pathways and platelet functions including degranulation, aggregation, thrombus formation, and spreading on fibrinogen. High serum levels of sJAM-A are associated with thrombo-ischemic complications in CAD. The study provides strong evidence for an atherosclerosis-promoting role of soluble JAM-A released from platelets.

## Data Availability

No datasets were generated or analysed during the current study.

## Abbreviations

apoE: Apolipoprotein E
CVD: Cardiovascular disease
EC: Endothelial cell
Ig: Immunoglobulin
JAM: Junctional adhesion molecule
miR: microRNA
oxLDL: Low density lipoprotein
sJAM-A: Soluble JAM-A
SMC: Smooth muscle cell
Tspan: Tetraspanin
